# Supplementary figures and images for: A host enzyme reduces metabolic dysfunction-associated steatotic liver disease (MASLD) by inactivating intestinal lipopolysaccharide
Source: eLife. 2025 Apr 24;13:RP100731. doi: 10.7554/eLife.100731 (PMC12021412; doi:10.7554/eLife.100731)

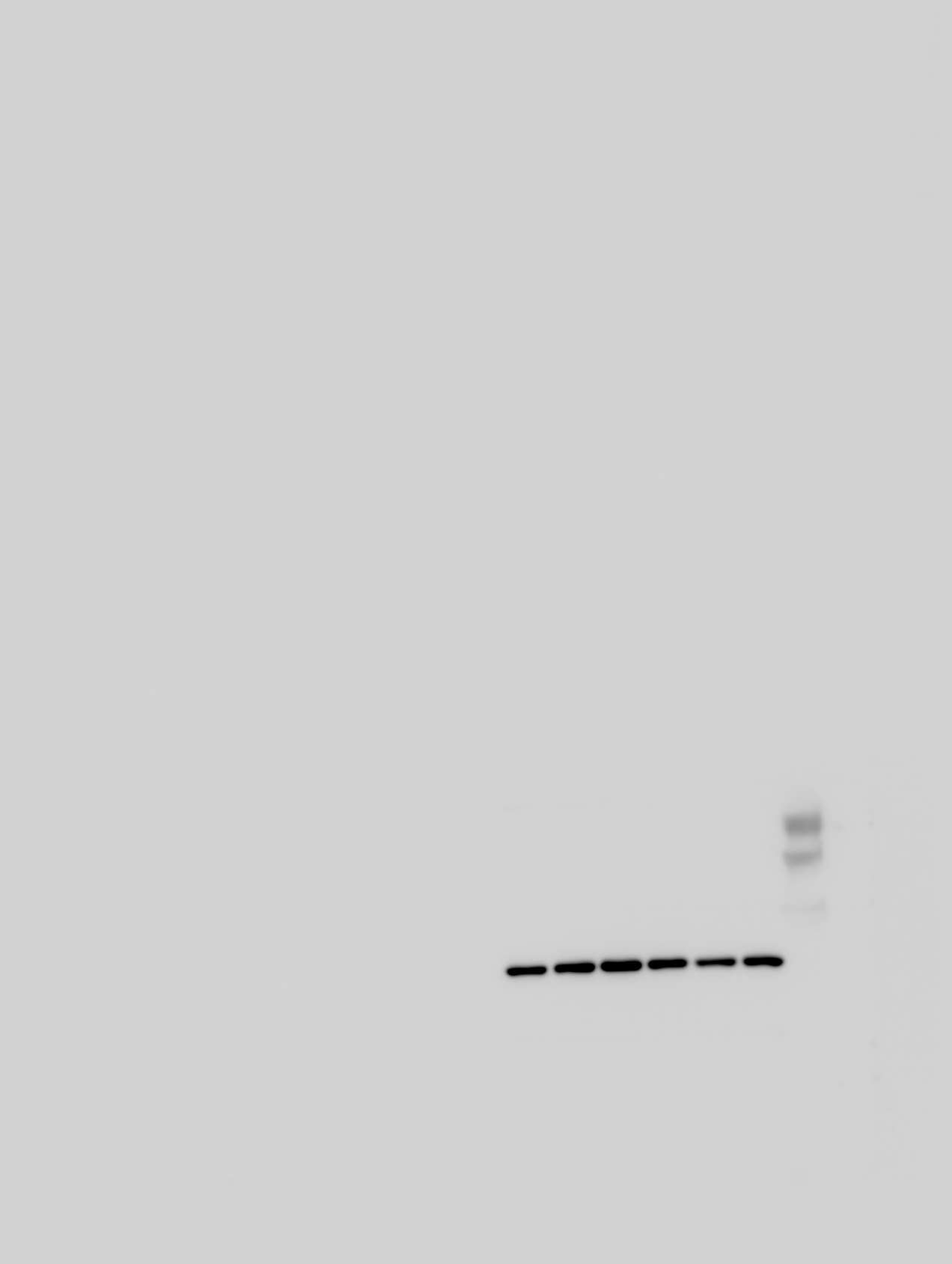

Supplement: Figure 5—source data 1. [file elife-100731-fig5-data1.zip › Figure 5í¬source data 1/Figure 5-source data 1 panels C LaminB.tif]

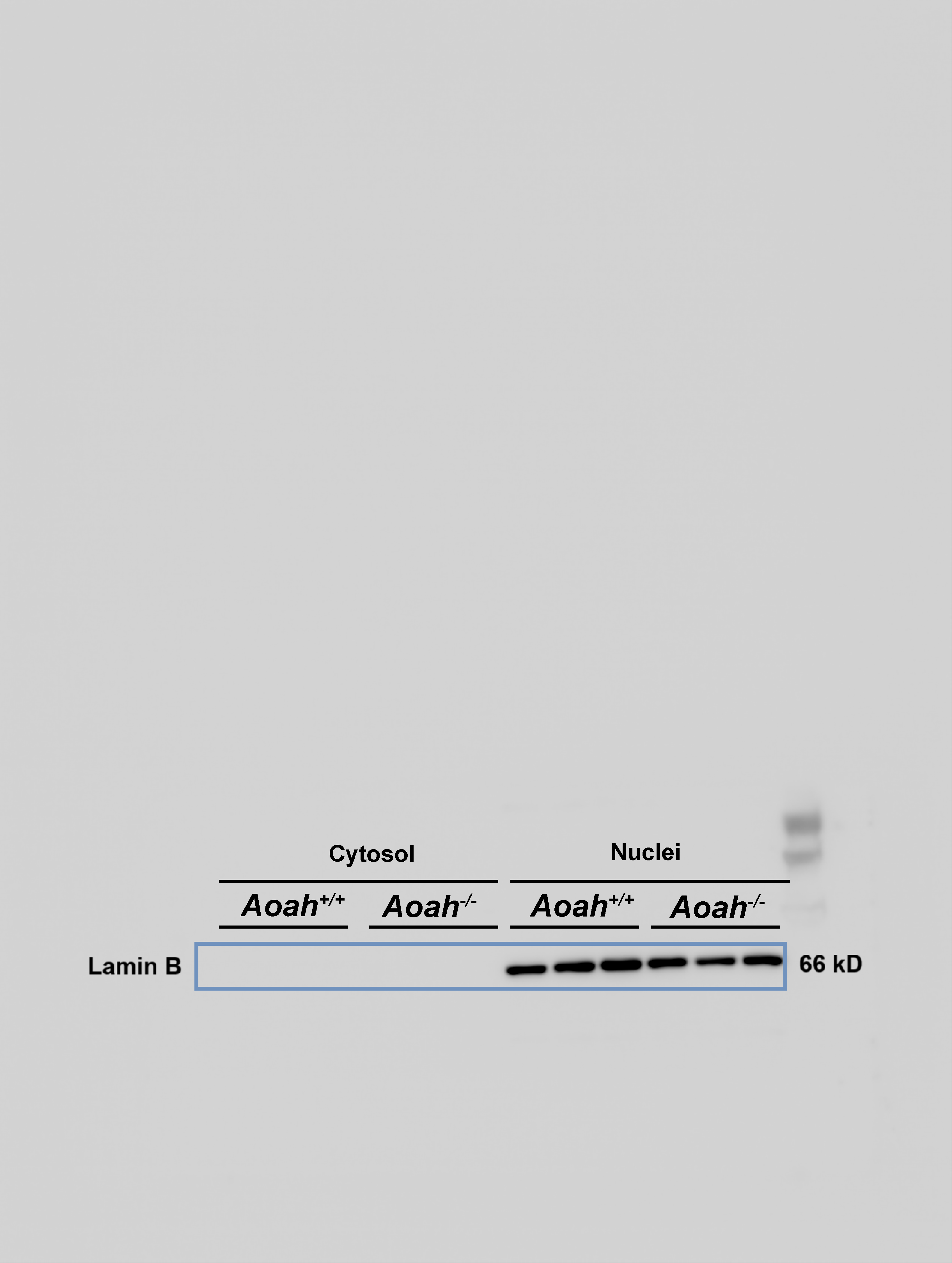

Supplement: Figure 5—source data 2. [file elife-100731-fig5-data2.zip › Figure 5í¬source data 2/Figure 5-source data 2 panels C LaminB.tif]

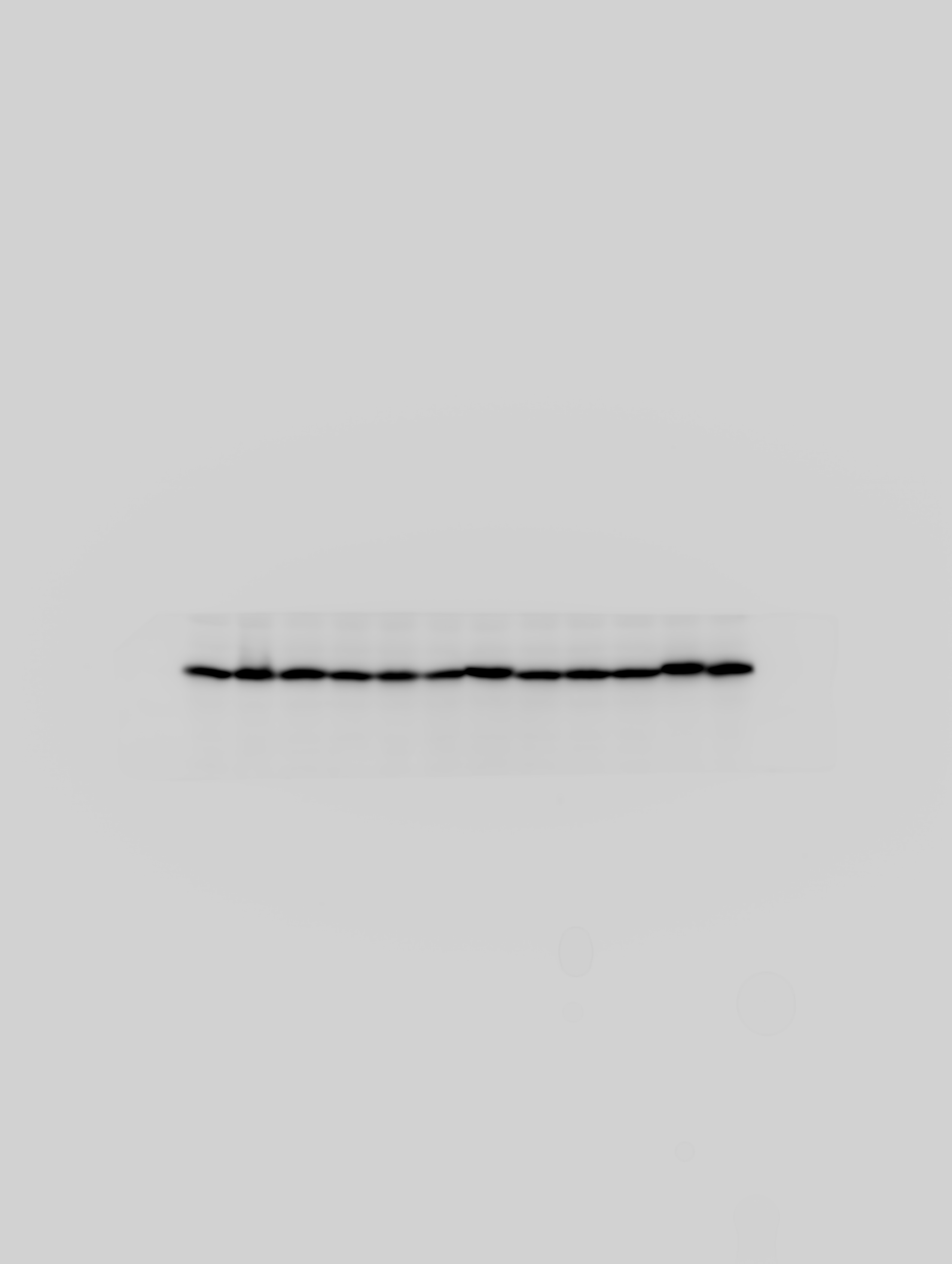

Supplement: Figure 6—source data 1. [file elife-100731-fig6-data1.zip › Figure 6í¬source data 1/Figure 6-source data 1 panels D S6.tif.tif]

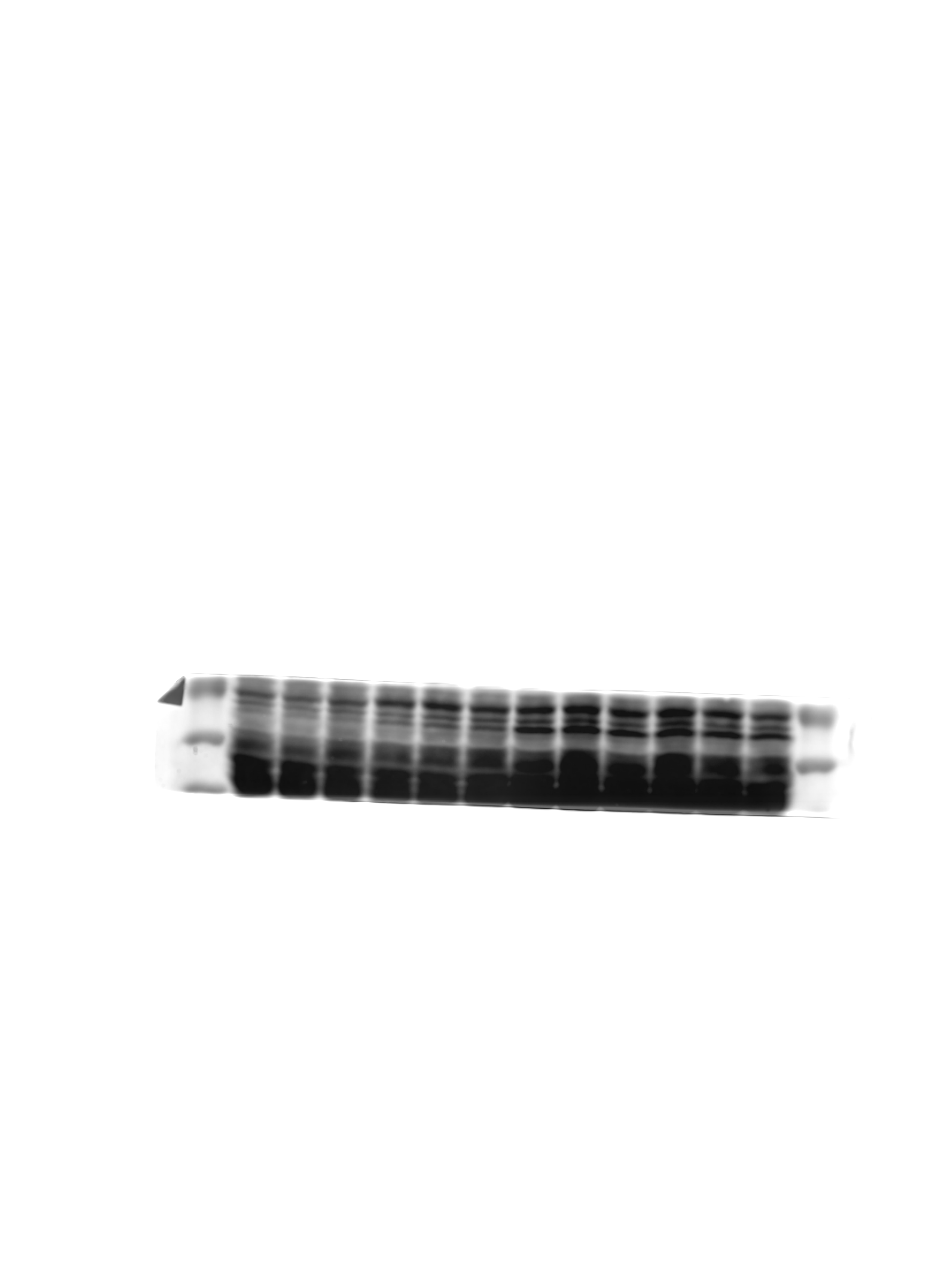

Supplement: Figure 6—source data 1. [file elife-100731-fig6-data1.zip › Figure 6í¬source data 1/Figure 6-source data 1 panels D pAKT.tif]

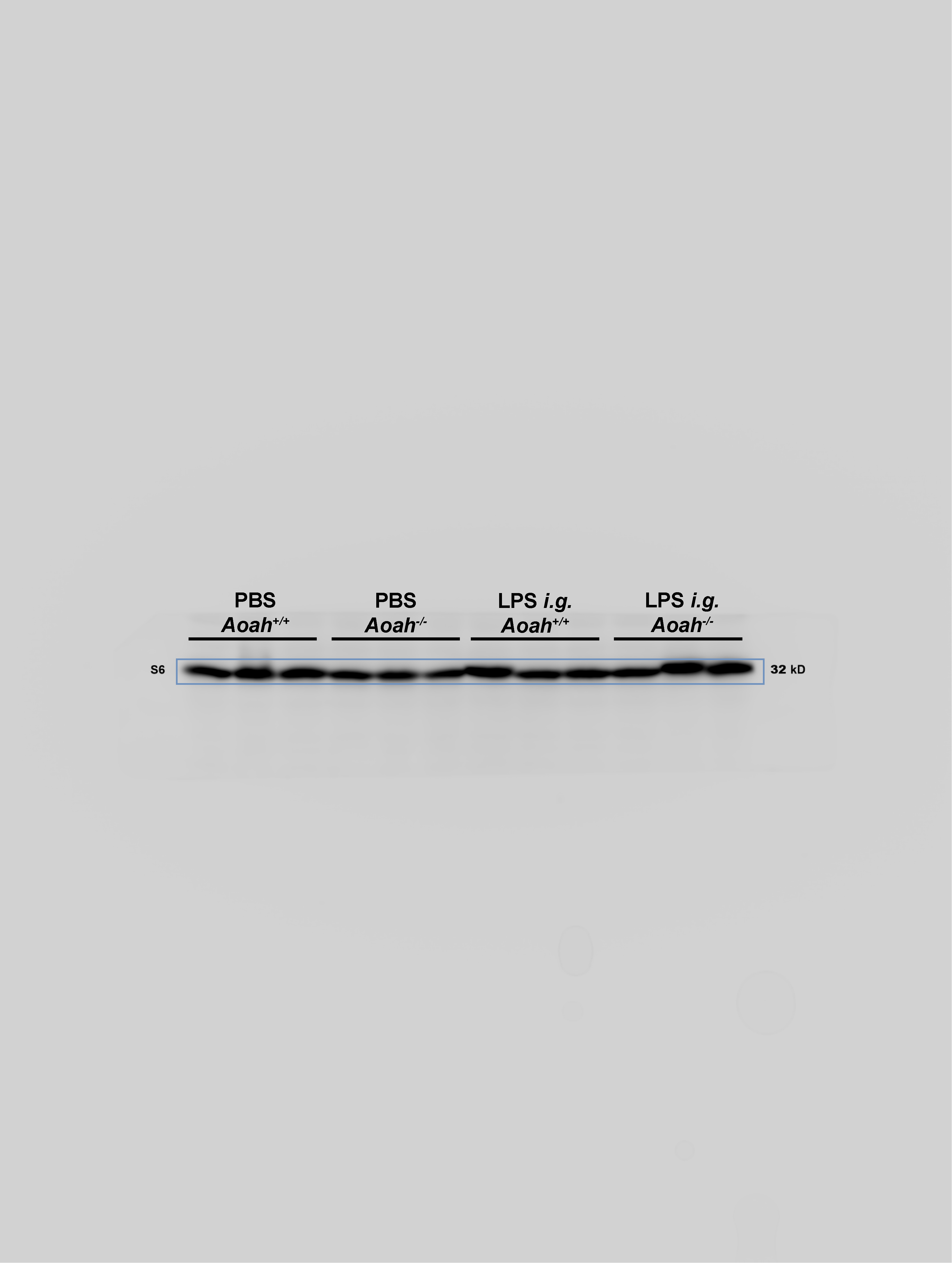

Supplement: Figure 6—source data 2. [file elife-100731-fig6-data2.zip › Figure 6í¬source data 2/Figure 6-source data 2 panels D S6.tif]

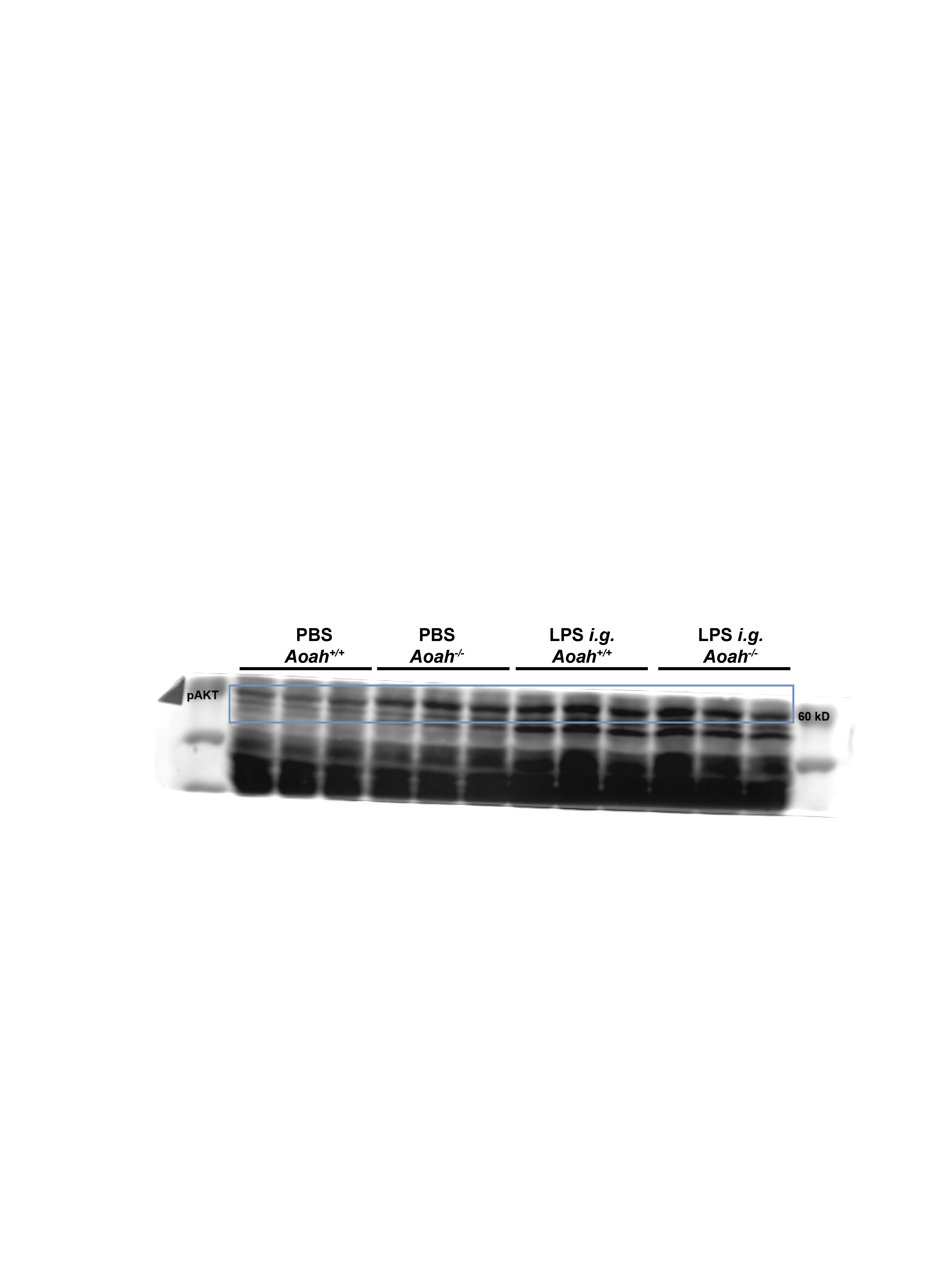

Supplement: Figure 6—source data 2. [file elife-100731-fig6-data2.zip › Figure 6í¬source data 2/Figure 6-source data 2 panels D pAKT.tif]
