# Supplementary material for: A host enzyme reduces metabolic dysfunction-associated steatotic liver disease (MASLD) by inactivating intestinal lipopolysaccharide
Source: eLife. 2025 Apr 24;13:RP100731. doi: 10.7554/eLife.100731 (PMC12021412; doi:10.7554/eLife.100731)
Supplement: Supplementary file 1. [file elife-100731-supp1.pdf]

Supplementary File 1. Primers used for qPCR

Mouse gene symbols	Forward primer sequence	Reverse primer sequence
Actb	5’-GGCTGTATTCCCCTCCATCG-3’	5’-CCAGTTGGTAACAATGCCATGT-3’
Il6	5’-ATCGTGGAAATGAGAAAAGAGTTGT-3’	5’-AAGTGCATCATCGTTGTTCATACA-3’
Tnfa	5’-CATCTTCTCAAAATTCGAGTGACAA-3’	5’-TCAGCCACTCCAGCTGCTC-3’
Ifng	5’-ATGAACGCTACACACTGCATC-3’	5’-CCATCCTTTTGCCAGTTCCTC-3’
Il10	5’-GCTGGACAACATACTGCTAACC-3’	5’-ATTTCCGATAAGGCTTGGCAA-3’
Timp1	5’-GCAACTCGGACCTGGTCATAA-3’	5’-CGGCCCGTGATGAGAAACT-3’
Mmp2	5’-CAAGTTCCCCGGCGATGTC-3’	5’-TTCTGGTCAAGGTCACCTGTC

		-3’
Acta	5’-TCCACCGCAAATGCTTCTAAG-3’	5’-TGTTGCTAGGCCAGGGCTAC-3’
Col4a1	5’-CTGGCACAAAAGGGACGAG-3’	5’-ACGTGGCCGAGAATTTCACC-3’
Cd36	5’-ATGGGCTGTGATCGGAACTG-3’	5’-GTCTTCCCAATAAGCATGTCTCC-3’
Fabp3	5’-ACCTGGAAGCTAGTGGACAG-3’	5’-TGATGGTAGTAGGCTTGGTCAT-3’
Fasn	5’-GGAGGTGGTGATAGCCGGTAT-3’	5’-TGGGTAATCCATAGAGCCCAG-3’
Acsl1	5’-TGCCAGAGCTGATTGACATTC-3’	5’-GGCATACCAGAAGGTGGTGAG-3’
Dgat2	5’-GCGCTACTTCCGAGACTACTT-3’	5’-GGGCCTTATGCCAGGAAACT-3’

Pnpla2	5’-GGATGGCGGCATTTCAGACA-3’	5’-CAAAGGGTTGGGTTGGTTCAG-3’
Pcx	5’-CTGAAGTTCCAAACAGTTCGAGG-3’	5’-CGCACGAAACACTCGGATG-3’
Acly	5’-AATCCTGGCTAAAACCTCGCC-3’	5’-GCATAGATGCACACGTAGAACT-3’
Acaca	5’-ATGGGCGGAATGG TCTCTTTC-3’	5’-TGGGGACCTTGTCTTCATCAT-3’
Acacb	5’-CCTTTGGCAACAAGCAAGGTA-3’	5’-AGTCGTACACATAGGTGGTCC-3’
Scd1	5’-GCCAGACCGGGCTGAACACC-3’	5’-GGCCTCCCAAGTGCAGCAGG-3’
Acss2	5’-AAACACGCTCAGGGAAAATCA-3’	5’-ACCGTAGATGTATCCCCCAGG-3’
Acot2	5’-CCCCAAGAGCATAGAAACCA-3’	5’-

		CCAATTCCAGGTCCTTTTACC-3’
Ppara	5’-AGAGCCCCATCTGTCCTCTC-3’	5’-ACTGGTAGTCTGCAAAACCAAA-3’
Aoah	5’-CAGCTACTCCCATGGCCAAA-3’	5’-GCCACCTGGACTGAAGAGTT-3’
Srebf1a	5’-GATGTGCGAACTGGACACAGC-3’	5’-GAGAAGCTCTCAGGAGAGTTGG-3’
Srebf1c	5’-CGCGGACCACGGAGCCATG-3’	5’-GAGAAGCTCTCAGGAGAGTTGG-3’
Saa1	5’-TTGTTCACGAGGCTTTCC-3’	5’-TGAGCAGCATCATAGTTCC-3’
Saa2	5’-TGGCTGGAAAGATGGAGACAA-3’	5’-AAAGCTCTCTCTTGCATCACTG-3’
Saa3	5’-TGCCATCATTCTTTGCATCTTGA-3’	5’-CCGTGAACTTCTGAACAGCCT-3’

Irak3	5'-TCCCACCTGAGGTGAAGCAT-3'	5'-TGTGACATTGGCTGGTTCCA-3'
